# Supplementary material for: Biparatopic sybodies neutralize SARS‐CoV‐2 variants of concern and mitigate drug resistance
Source: EMBO Rep. 2022 Mar 7;23(4):e54199. doi: 10.15252/embr.202154199 (PMC8982573; doi:10.15252/embr.202154199)
Supplement: Supplementary file 2 — Appendix [file EMBR-23-e54199-s002.pdf]

Supplementary Material

Table of content

Appendix Tables S1 and S2, Pages 2-4

Appendix Figures S1-S4, Pages 5-8

Appendix Table S1 – Sybody protein sequences

**Sb#14**	QVQLVESGGGLVQAGGSLRLSCAASGFPVQAREMEWYRQAPGKEREWVAAIKSTGTYTAYAYSVK GRFTISRDNAKNTVYLQMNSLKPEDTAVYYCYVYVGSSYIGQGTQVTVS
**Sb#15**	QVQLVESGGGLVQAGGSLRLSCAASGFPVKNFEMEWYRKAPGKEREWVAAIQSGGVETYYADSVK GRFTISRDNAKNTVYLQMNSLKPEDTAVYYCFVYVGRSYIGQGTQVTVS
**Sb#16**	QVQLVESGGGLVQAGGSLRLSCAASGFPVAYKTMWWYRQAPGKEREWVAAIESYGIKWTRYADSV KGRFTISRDNAKNTVYLQMNSLKPEDTAVYYCIVWVGAQYHGQGTQVTVS
**Sb#42**	QVQLVESGGGLVQAGGSLRLSCAASGFPVYWHHMHWYRQAPGKEREWVAAIISWGWYTTYADSVK GRFTISRDNAKNTVYLQMNSLKPEDTAVYYCNVKDHGAQNQMYDYWGQGTQVTVS
**Sb#45**	QVQLVESGGGLVQAGGSLRLSCAASGFPVYRDRMAWYRQAPGKEREWVAAIYSAGQQTRYADSVK GRFTISRDNAKNTVYLQMNSLKPEDTAVYYCNVKDVGHHYEYYDYWGQGTQVTVS
**Sb#68**	QVQLVESGGGSVQAGGSLRLSCAASGSISSITYLGWFRQAPGKEREGVAALITVNGHTYYADSVK GRFTVSLDNAKNTVYLQMNSLKPEDTALYYCAAAAWGYAWPLHQDDYWYWGQGTQVTVS

Appendix Table S2 – Cryo-EM data collection, refinement and validation statistics

	**S-2P + Sb#15 + Sb#68**		**S-2P + Sb#15**	**S-6P + Sb#68**	
	3up (EMD-12082, PDB 7P77)	*1up/1up-out/1down* (EMD-12083, PDB 7P78)	*1up/1up-out/1down* (EMD-12084, PDB 7P79)	*2up/1flexible* (EMD-12085, PDB 7P7A)	*1up/2down* (EMD-12086, PDB 7P7B)
**Data collection and processing**					
Magnification	49,407	49,407	49,407	49,407	49,407
Voltage (kV)	200	200	200	200	200
Electron exposure (e–/Å^2)	53	53	53	53	53
Defocus range (μm)	-0.9 to -1.9	-0.9 to -1.9	-0.9 to -1.9	-0.9 to -1.9	-0.9 to -1.9
Pixel size (Å)	1.012	1.012	1.012	1.012	1.012
Symmetry imposed	C3	C1	C1	C1	C1
Initial particle images (no.)	637,105	637,105	66,632	344,976	344,976
Final particle images (no.)	78,933	52,839	22,055	24,325	84,917
Map resolution (Å)	2.98	3.32	4.04	4.76	3.13
FSC threshold	0.143				
Map resolution range (Å)	2.5 – 8.5	2.8 – 10.6	3.7-16.6	4.4-23.5	2.7-9.8
**Refinement**					
Initial model used	PDB 7MY2	PDB 7MY2	PDB 7MY2	PDB 7MY2	PDB 6ZGG
Model resolution (Å) (0.5 FSC threshold)	3.0	3.3	4.0	4.8	3.1
Model resolution range (Å)	15-3.0	15-3.3	17-4.0	24-4.8	15-3.1
Map sharpening *B* factor (Å^2)	-99	-82.7	-80.4	-118.3	-92.2
Model composition					
Nonhydrogen atoms	30582	28807	26907	25421	25096

Protein residues	3822	3625	3377	3159	3207
Ligands	66	56	56	65	0
B factors (Å^2)					
Protein	129.92	220.07	121.98	195.00	185.59
Ligand	114.69	157.41	138.65	217.68	-
R.m.s. deviations					
Bond lengths (Å)	0.006	0.006	0.007	0.007	0.006
Bond angles (°)	1.059	1.078	1.220	1.190	1.005
Validation					
MolProbity score	1.47	1.54	1.59	1.59	1.43
Clashscore	3.61	4.39	4.23	4.71	3.07
Poor rotamers (%)	0.00	0.00	0.00	0.00	0.04
Ramachandran plot					
Favored (%)	95.46	95.39	94.39	95.09	95.24
Allowed (%)	4.54	4.58	5.61	4.95	4.73
Disallowed (%)	0.00	0.03	0.00	0.00	0.03

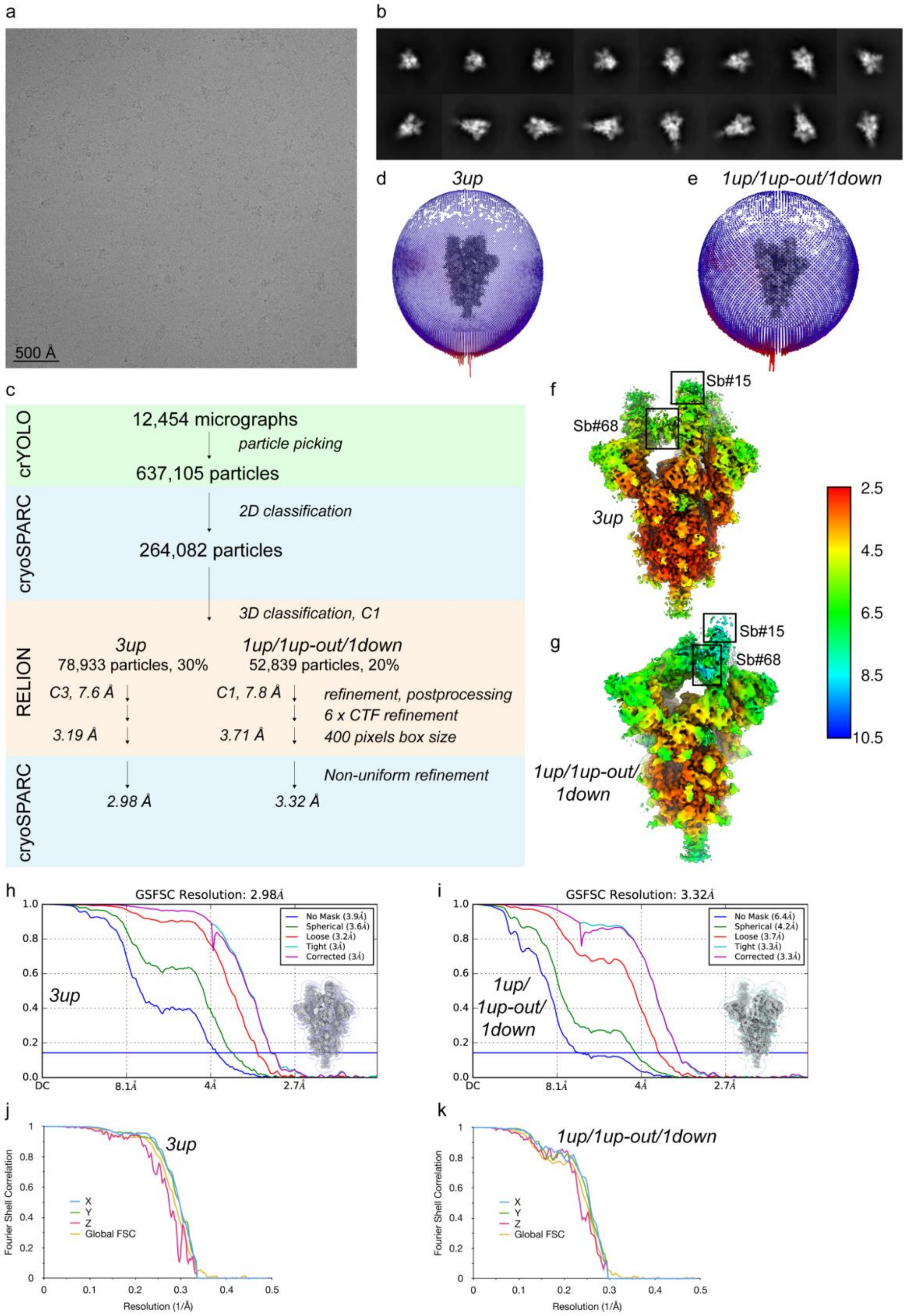

Appendix Figure S1

Cryo-EM reconstruction of S-2P in complex with Sb#15 and Sb#68. Representative cryo-EM image (**A**) and 2D-class averages (**B**) of vitrified S-2P in the presence of both Sb#15 and Sb#68. (**C**) Image processing work flow. Angular particle distribution plot (**D** and **E**), final reconstructed map colored by local resolution, as estimated in cryoSPARC (**F** and **G**), FSC plot (**H** and **I**) and anisotropy plot used for resolution estimation (**J** and **K**) for the final 3*up* and 1*up*/1*up-out*/1*down* RBD reconstruction, respectively. (**H** and **I**) The line indicates the FSC thresholds used for FSC of 0.143, and the mask used for FSC calculation overlaid on the map is shown as thumbnail. (**J** and **K**) The global FSC curve is represented in yellow, while the directional FSCs along the x, y and z axis are displayed in blue, green and red, respectively.

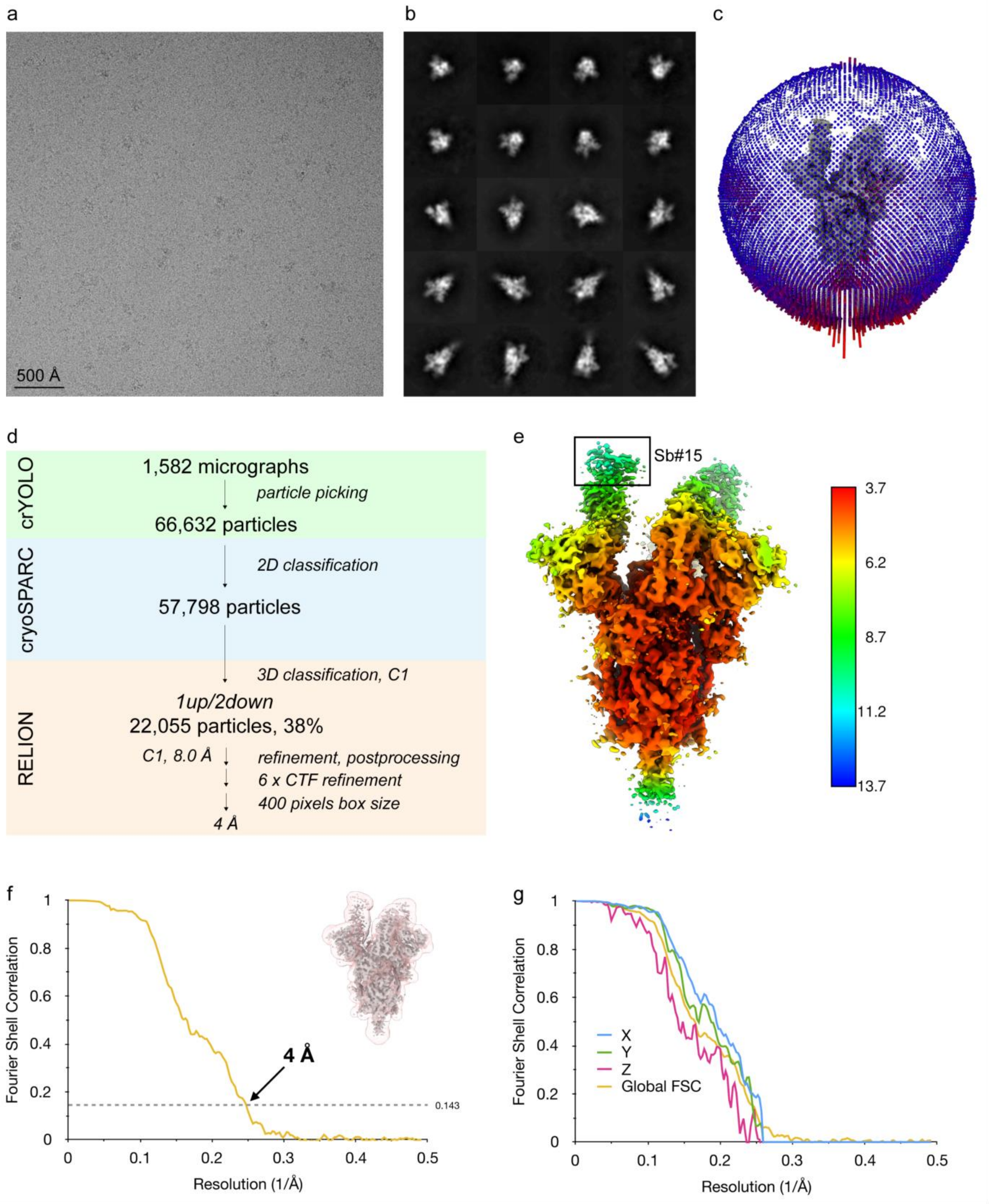

Appendix Figure S2

Cryo-EM reconstruction of S-2P in presence of Sb#15. Representative cryo-EM image (**A**) and 2D-class averages (**B**) of vitrified S-2P in the presence of Sb#15. (**C**) Angular distribution plot of particles included in the final map. (**D**) Image processing work flow. Final reconstructed map colored by local resolution, as estimated in RELION (**E**), FSC plot (**F**) and anisotropy plot used for resolution estimation (**G**) for the final 1*up*/2*down* RBD reconstruction. (**F**) The line indicates the FSC thresholds used for FSC of 0.143, and the mask used for FSC calculation overlaid on the map is shown as thumbnail. (**G**) The global FSC curve is represented in yellow, while the directional FSCs along the x, y and z axis are displayed in blue, green and red, respectively.

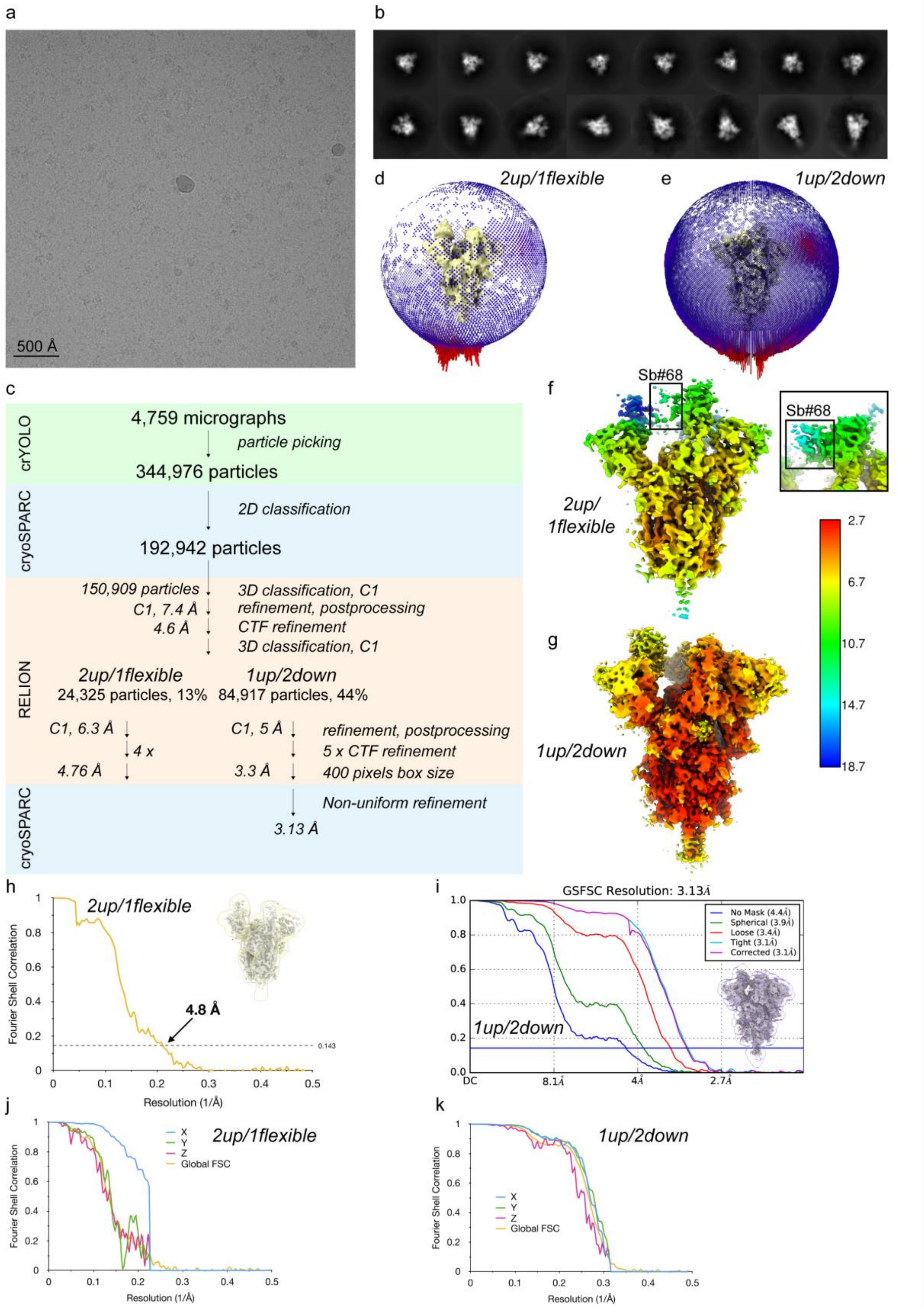

Appendix Figure S3

Cryo-EM reconstruction of S-6P in presence of Sb#68. Representative cryo-EM image (**A**) and 2D-class averages (**B**) of vitrified S-6P in the presence of Sb#68. (**C**) Image processing work flow. Angular particle distribution plot (**D** and **E**), final reconstructed map colored by local resolution, as estimated in cryoSPARC and RELION (**F** and **G**), FSC plot (**H** and **I**) and anisotropy plot used for resolution estimation (**J** and **K**) for the final *2up/1flexible* and 1*up/2down* RBD reconstruction, respectively. (**H** and **I**) The line indicates the FSC thresholds used for FSC of 0.143, and the mask used for FSC calculation overlaid on the map is shown as thumbnail. (**J** and **K**) The global FSC curve is represented in yellow, while the directional FSCs along the x, y and z axis are displayed in blue, green and red, respectively.

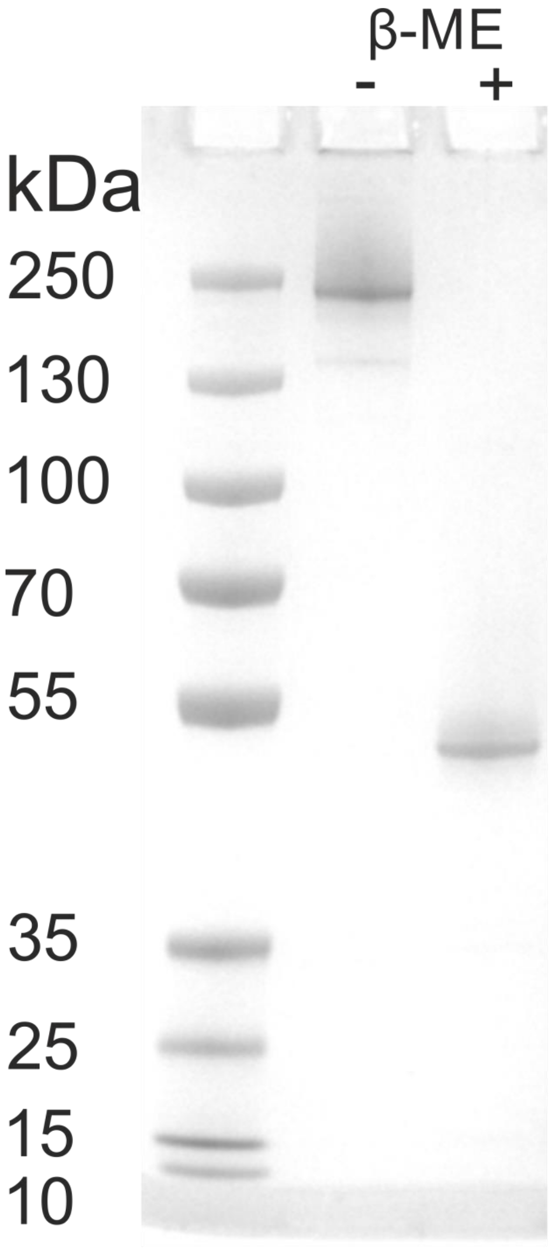

Appendix Figure S4

SDS-PAGE analysis of Tripod-GS4r. Purified Tripod-GS4r was loaded on a SDS-PAGE gel with and without incubation of β-mercaptoethanol (β-ME) and stained using coomassie blue.
