# Supplementary material for: Biparatopic sybodies neutralize SARS‐CoV‐2 variants of concern and mitigate drug resistance
Source: EMBO Rep. 2022 Mar 7;23(4):e54199. doi: 10.15252/embr.202154199 (PMC8982573; doi:10.15252/embr.202154199)
Supplement: Supplementary file 3 — Expanded View Figures PDF [file EMBR-23-e54199-s003.pdf]

Expanded View Figures

Figure EV1. Kinetic characterization of sybodies by GCI.

A RBD-vYFP and ECD were immobilized as indicated and the six top sybodies were injected at increasing concentrations ranging from 1.37 nM to 1 μM. Data were fitted using a Langmuir 1:1 model.

B In-depth affinity characterization of Sb#15 and Sb#68. RBD-vYFP and S-6P were immobilized as indicated and Sb#15 and Sb#68 were injected at concentrations ranging from 1.95 to 250 nM for Sb#15 and 3.9 to 500 nM for Sb#68. For RBD, data were fitted using a Langmuir 1:1 model. For S-6P, the data were fitted with the heterogeneous ligand model, because the 1:1 model was clearly not appropriate to describe the experimental data. Corresponding data for S-2P is shown in main Fig 1A.

 © 2022 The Authors

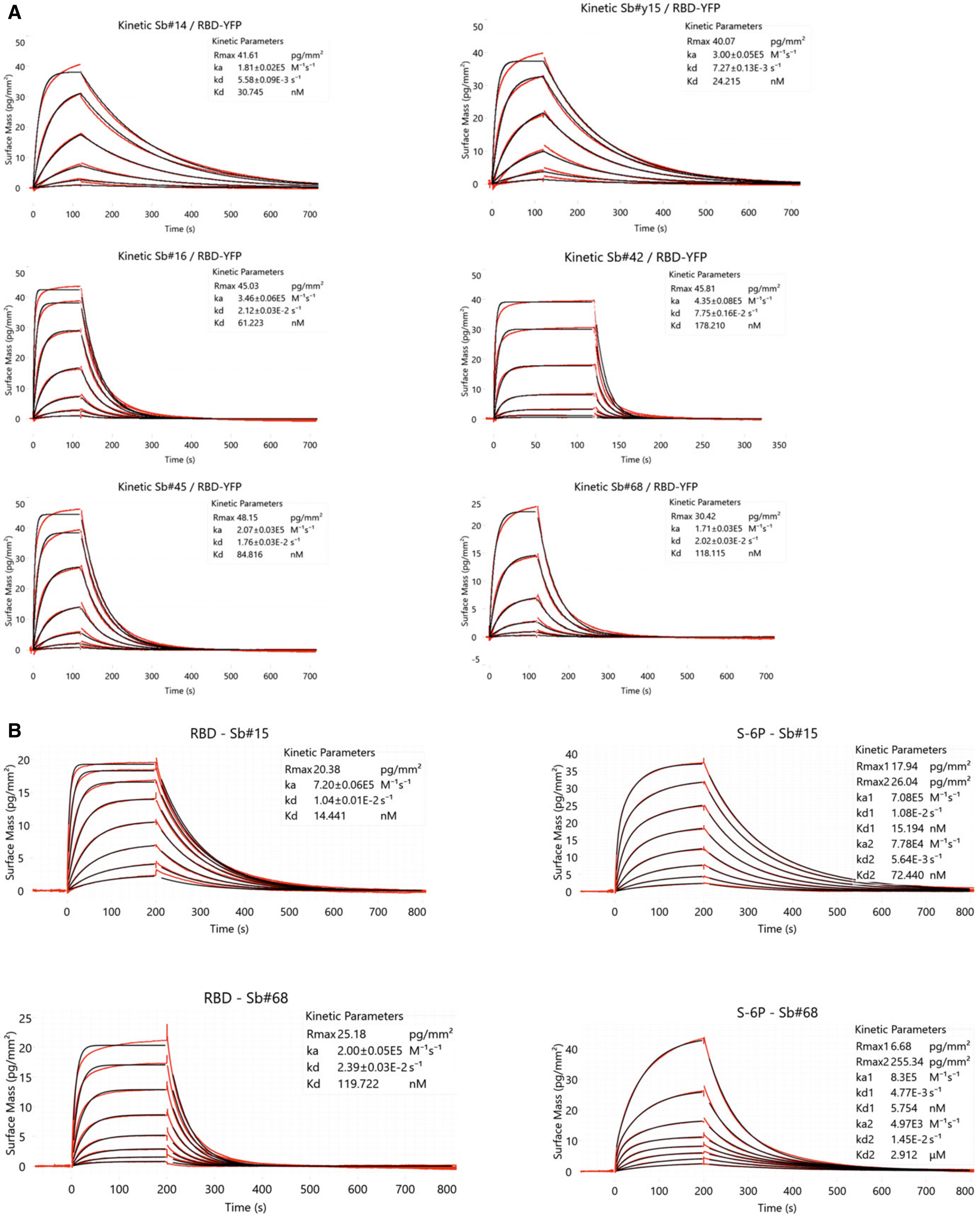

A
Kinetic Sb#14 / RBD-YFP
Kinetic Parameters
Rmax 41.61 pg/mm²
ka 1.81±0.02E5 M⁻¹s⁻¹
kd 5.58±0.09E-3 s⁻¹
Kd 30.745 nM
Kinetic Sb#y15 / RBD-YFP
Kinetic Parameters
Rmax 40.07 pg/mm²
ka 3.00±0.05E5 M⁻¹s⁻¹
kd 7.27±0.13E-3 s⁻¹
Kd 24.215 nM
Kinetic Sb#16 / RBD-YFP
Kinetic Parameters
Rmax 45.03 pg/mm²
ka 3.46±0.06E5 M⁻¹s⁻¹
kd 2.12±0.03E-2 s⁻¹
Kd 61.223 nM
Kinetic Sb#42 / RBD-YFP
Kinetic Parameters
Rmax 45.81 pg/mm²
ka 4.35±0.08E5 M⁻¹s⁻¹
kd 7.75±0.16E-2 s⁻¹
Kd 178.210 nM
Kinetic Sb#45 / RBD-YFP
Kinetic Parameters
Rmax 48.15 pg/mm²
ka 2.07±0.03E5 M⁻¹s⁻¹
kd 1.76±0.03E-2 s⁻¹
Kd 84.816 nM
Kinetic Sb#68 / RBD-YFP
Kinetic Parameters
Rmax 30.42 pg/mm²
ka 1.71±0.03E5 M⁻¹s⁻¹
kd 2.02±0.03E-2 s⁻¹
Kd 118.115 nM
Surface Mass (pg/mm²)
Time (s)
B
RBD - Sb#15
Kinetic Parameters
Rmax 20.38 pg/mm²
ka 7.20±0.06E5 M⁻¹s⁻¹
kd 1.04±0.01E-2 s⁻¹
Kd 14.441 nM
S-6P - Sb#15
Kinetic Parameters
Rmax1 17.94 pg/mm²
Rmax2 26.04 pg/mm²
ka1 7.08E5 M⁻¹s⁻¹
kd1 1.08E-2 s⁻¹
Kd1 15.194 nM
ka2 7.78E4 M⁻¹s⁻¹
kd2 5.64E-3 s⁻¹
Kd2 72.440 nM
RBD - Sb#68
Kinetic Parameters
Rmax 25.18 pg/mm²
ka 2.00±0.05E5 M⁻¹s⁻¹
kd 2.39±0.03E-2 s⁻¹
Kd 119.722 nM
S-6P - Sb#68
Kinetic Parameters
Rmax1 6.68 pg/mm²
Rmax2 255.34 pg/mm²
ka1 8.3E5 M⁻¹s⁻¹
kd1 4.77E-3 s⁻¹
Kd1 5.754 nM
ka2 4.97E3 M⁻¹s⁻¹
kd2 1.45E-2 s⁻¹
Kd2 2.912 μM
Surface Mass (pg/mm²)
Time (s)

Figure EV1.

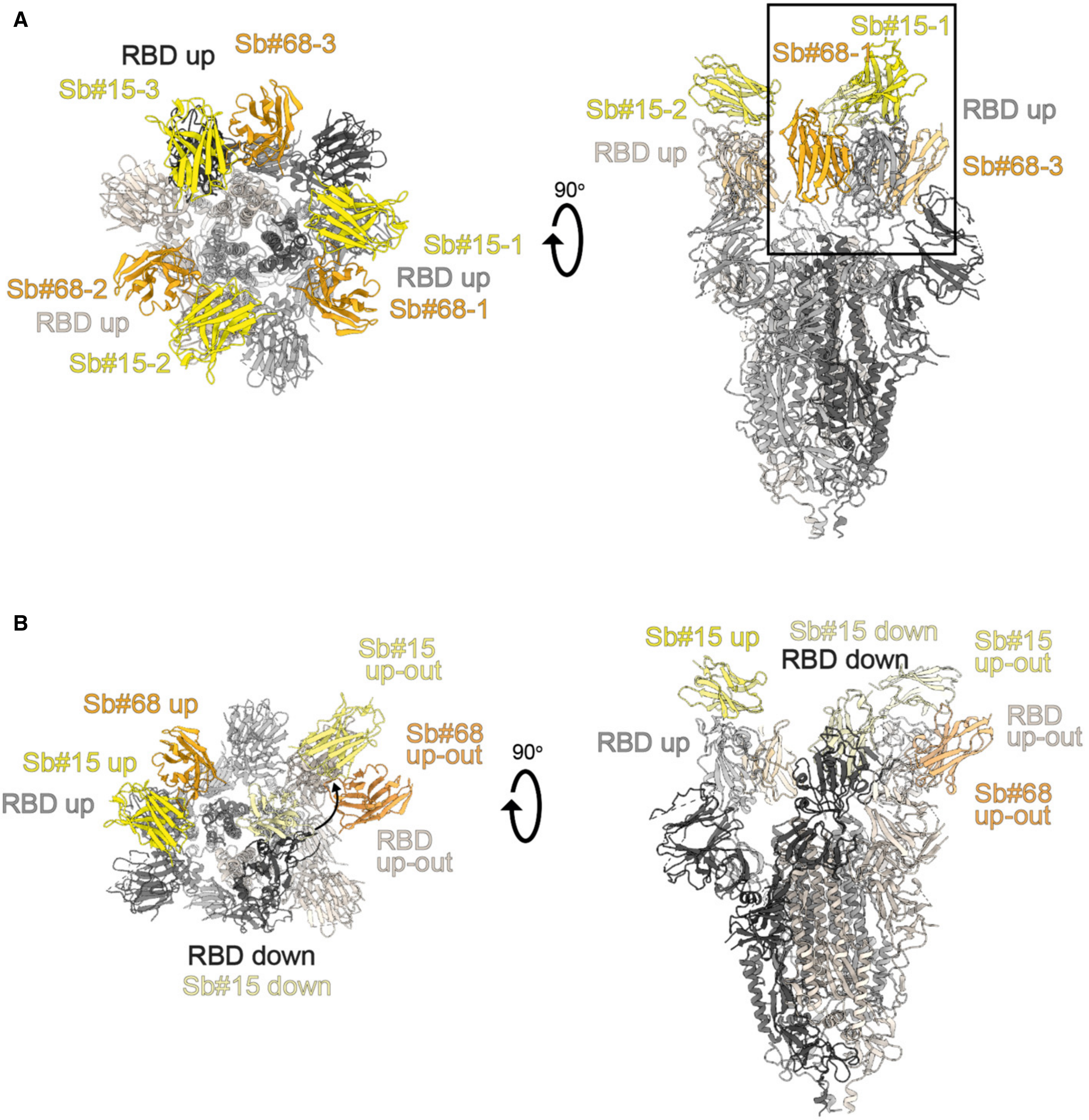

Figure EV2. Structures of S-2P spike in complex with both Sb#15 and Sb#68.

A Structure of S-2P with both Sb#15 and Sb#68 bound to each RBD adopting a symmetrical *3up* conformation. Based on the cryo-EM map shown in main Fig 5A, a model shown as ribbon was built using pre-existing structures (PDB ID:6X2B for S-2P; PDB ID:3K1K for Sb#15; PDB ID:5M13 for Sb#68).

B Structure of S-2P with the three RBDs adopting an asymmetrical *1up/1up-out/1down* conformation. Based on the cryo-EM map shown in Fig 5B, a model shown as ribbon was built using pre-existing structures (PDB ID:6X2B for S-2P; PDB ID:3K1K for Sb#15; PDB ID:5M13 for Sb#68). The *up-out* state is pushed outward by the adjacent RBD in a *down* state with bound Sb#15 (arrow). Spike protein is shown in shades of gray, Sb#15 in yellow, and Sb#68 in orange.

 © 2022 The Authors

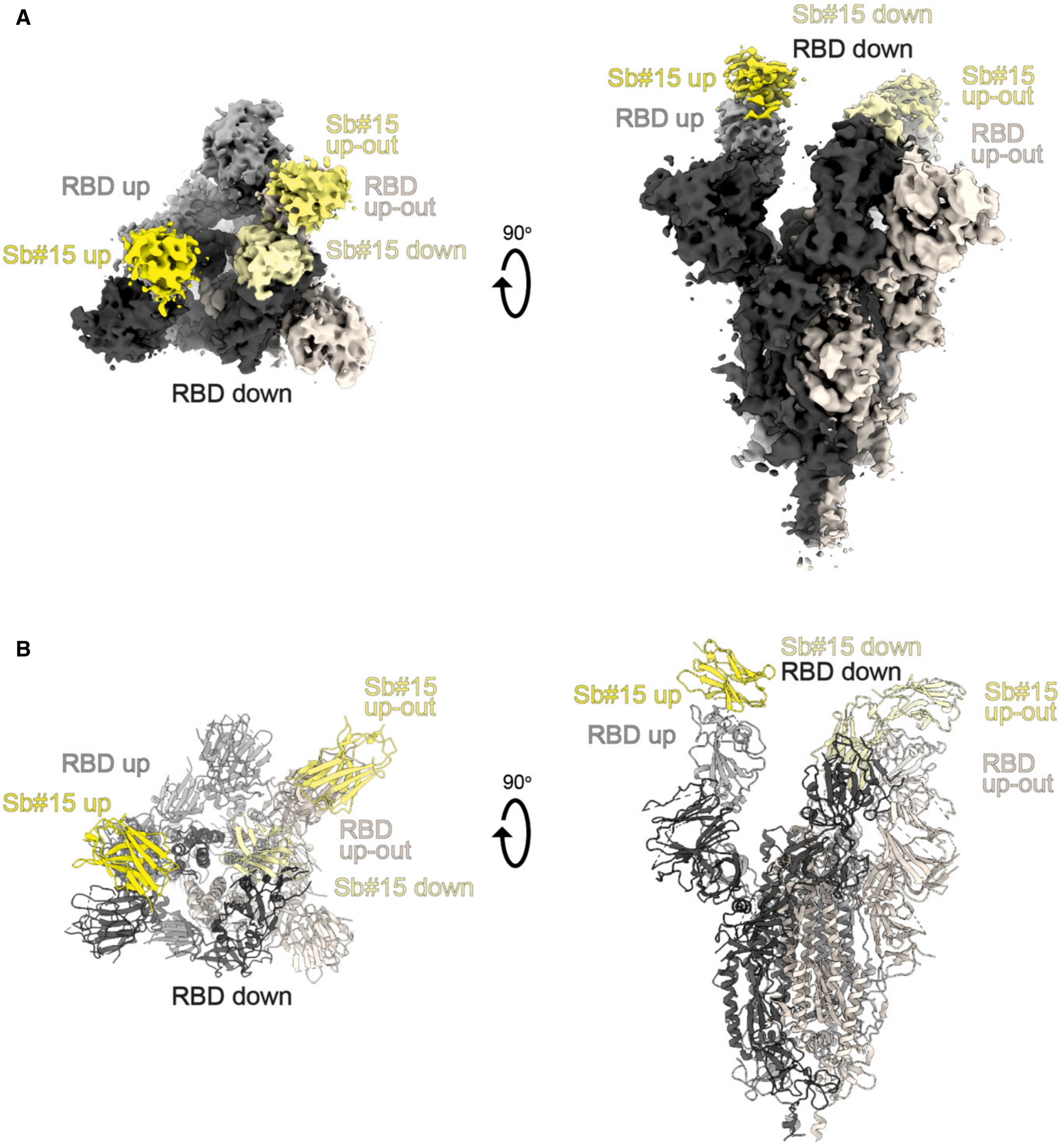

Figure EV3. Structural analysis of the S-2P/Sb#15 complex.

A Cryo-EM map of S-2P with Sb#15 bound to each RBD adopting an asymmetrical *1up/1up-out/1down* conformation.

B The corresponding model built using pre-existing structures (PDB ID:6X2B for S-2P; PDB ID:3K1K for Sb#15) is shown as ribbon. Final map blurred to a B factor of −30 Å was used for better clarity of the less resolved RBDs and sybodies. Spike protein is shown in shades of gray and Sb#15 in yellow.

© 2022 The Authors

Figure EV4. Structural analysis of S-2P/Sb#68 complex.

A Cryo-EM map of S-6P, with an *1up/2down* RBD conformation.

B The corresponding model is shown as ribbon (PDB ID:6ZGG for S-6P). No densities for Sb#68 were observed.

C Cryo-EM map of S-6P, with two Sb#68 bound to *up*-RBDs of the spike featuring a *2up/1flexible* conformation.

D The corresponding model built on pre-existing structures (PDB ID:6X2B for S-6P; PDB ID:5M13 for Sb#68) is shown as ribbon.

E Sb#68 cannot bind to *up*-RBD if the neighboring RBD exhibits a *down* conformation due to steric clashing. Final maps blurred to a B factor of −30 Å were used for better clarity of the less resolved RBDs and sybodies. Spike protein is shown in shades of gray and Sb#68 in orange.

 © 2022 The Authors

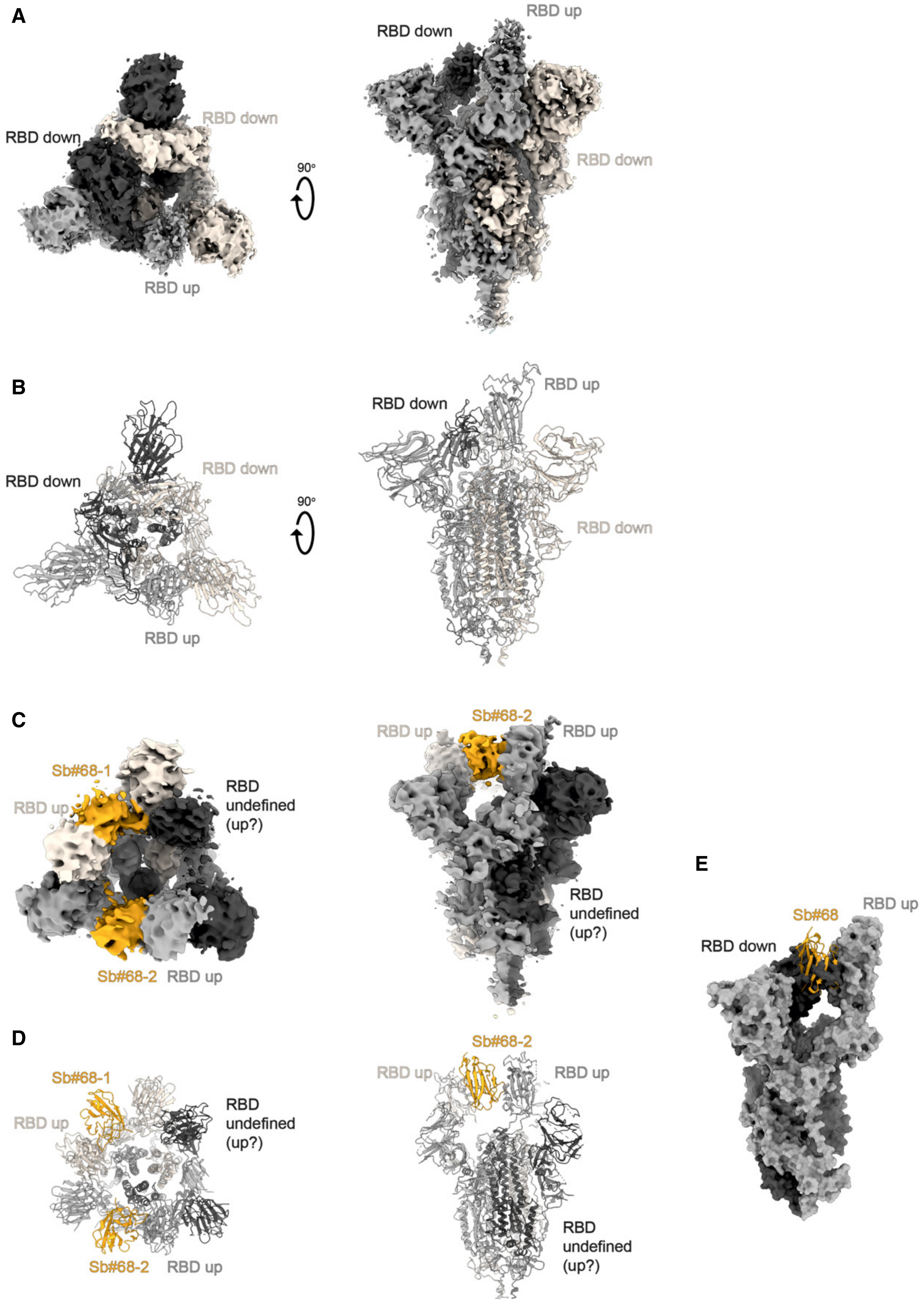
A
RBD down
RBD down
RBD up
90°
RBD down
RBD up
RBD down
B
RBD down
RBD down
RBD up
90°
RBD down
RBD up
RBD down
C
Sb#68-1
RBD up
RBD
undefined
(up?)
Sb#68-2
RBD up
RBD up
Sb#68-2
RBD up
RBD
undefined
(up?)
D
Sb#68-1
RBD up
RBD
undefined
(up?)
Sb#68-2
RBD up
Sb#68-2
RBD up
RBD up
RBD
undefined
(up?)
E
Sb#68
RBD up
RBD down

Figure EV4.

Figure EV5. Antibody, nanobody, and sybody binding to RBD epitopes.

A Schematic representation of the ACE2 binding site and the cryptic epitope on the RBD.

B Sb#15 binds to the top of RBD (the ACE2 epitope) and Sb#68 binds to the side of the RBD and recognizes a conserved cryptic epitope.

C Superposition of the structures of binders recognizing the cryptic epitope: EY6A (PDB ID: 6ZCZ), CR3022 (PDB ID: 6W41), Nb12 (PDB ID:7MY3), Nb30 (PDB ID:7MY2), VHH-U (PDB ID: 7KN5), VHH-V (PDB ID: 7KN6), VHH-W (PDB ID: 7KN7), NM1226 (PDB ID: 7NKT), and WNb10 (PDB ID: 7LX5).

D Superposition of the structures of binders recognizing the ACE2 epitope: WNb2 (PDB ID: 7LDJ), H11-D4 (PDB ID: 6YZ5), SR4 (PDB ID: 7C8V), Nb20 (PDB ID: 7JVB), VHH E (7KN5), NM1230 (PDB ID: 7B27), and MR17m (PDB ID: 7C8W). Structures are shown as surfaces.

 © 2022 The Authors

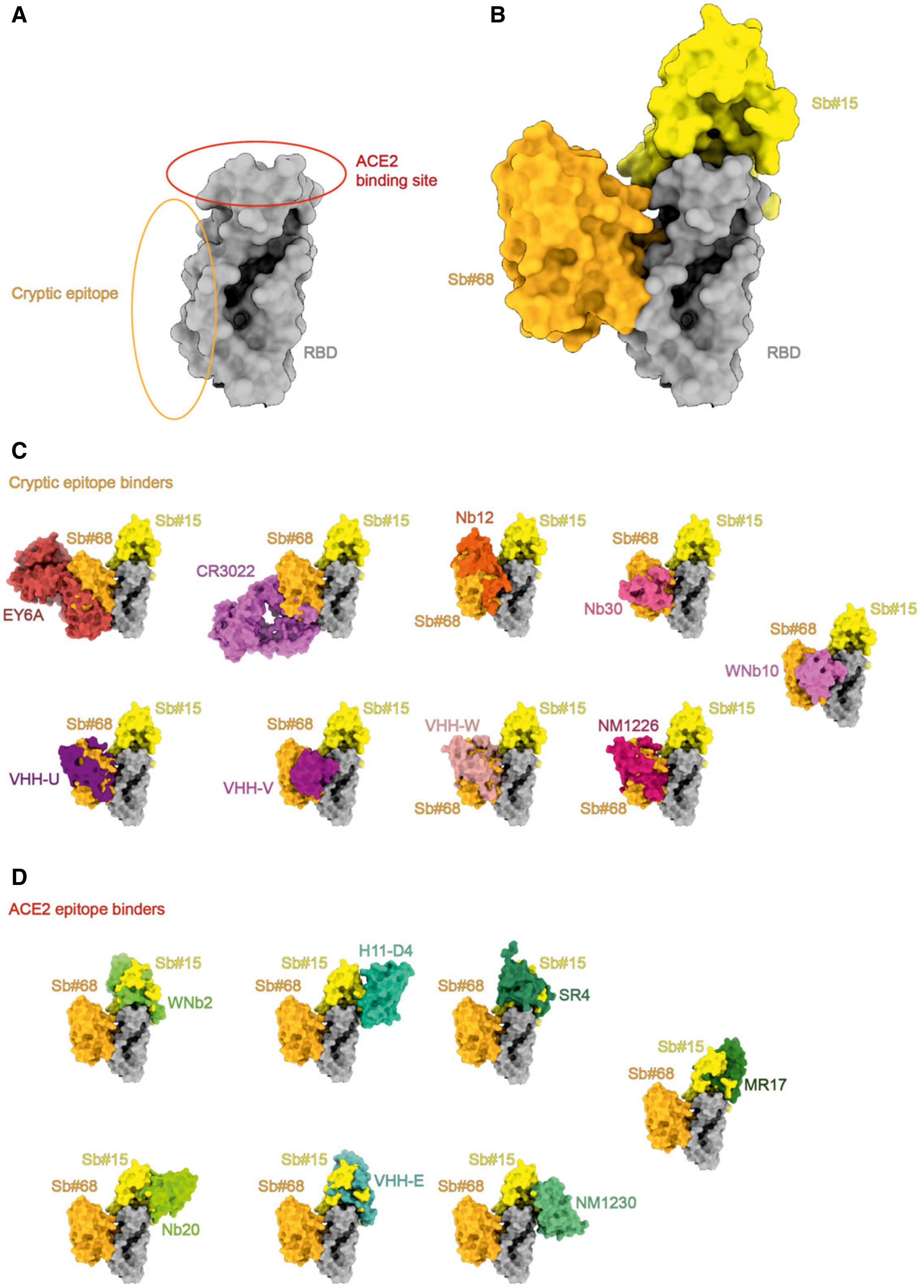
A
ACE2
binding site
Cryptic epitope
RBD
B
Sb#15
Sb#68
RBD
C
Cryptic epitope binders
Sb#68
Sb#15
EY6A
CR3022
Nb12
Nb30
WNb10
VHH-U
VHH-V
VHH-W
NM1226
D
ACE2 epitope binders
WNb2
H11-D4
SR4
MR17
Nb20
VHH-E
NM1230

Figure EV5.
